# Significance of HE4 estimation in comparison with CA125 in diagnosis of ovarian cancer and assessment of treatment response

**DOI:** 10.1186/1746-1596-8-11

**Published:** 2013-01-23

**Authors:** Elham O Hamed, Hydi Ahmed, Osama B Sedeek, Abeer M Mohammed, Ali A Abd-Alla, Hazem M Abdel Ghaffar

**Affiliations:** 1 Clinical Pathology Department, Faculty of Medicine, Sohag University, Sohag, 82524, Egypt; 2 Clinical Oncology Department, Faculty of Medicine, Sohag University, Sohag, 82524, Egypt; 3 Obstetrics & Gynecology Department, Faculty of Medicine, Sohag University, Sohag, Egypt; 4 Clinical Pathology Department, Faculty of Medicine, Assiut University, Assiut, Egypt

**Keywords:** Human epididymis protein 4, HE4, Carbohydrate antigen125, CA125, Epithelial, Ovarian cancer, EOC

## Abstract

**Background:**

Human epididymis protein 4 (HE4) is a novel and specific biomarker for ovarian cancer. The aim of this study is to evaluate a new tumor marker, HE4, in comparison with CA125 in diagnosis of epithelial ovarian cancer (EOC) and benign gynecological diseases.

**Methods:**

CA125 and HE4 serum levels were determined in 30 patients with epithelial ovarian cancer (21 serous, 6 endometrioid and 3 mucinous tumors), 20 patients with benign gynecological diseases (8 patients with ovarian cyst, 5 patients with endometriosis, 4 patients with fibroid and 3 patients with pelvic inflammatory disease) and 20 healthy women. CA125 and HE4 cut-offs were 35 U/ml and 150 pmol/l, respectively.

**Results:**

Serum HE4 and CA125 concentrations were significantly higher in the ovarian cancer patients compared with those seen in patients with benign disease or in the healthy controls (p = 0.001 and p < 0.001 respectively). In the receiver operating characteristic analysis (ROC), the area under the curve (AUC) values for HE4 was 0.96 (95% confidence interval, 0.9-1.0) and CA125 was 0.82 (95% confidence interval, 0.7-0.94). Compared to CA125, HE4 had higher sensitivity (90% vs. 83.3%), specificity (95% vs. 85%), PPV (93.1% vs. 80.7%) and NPV (92.7% vs. 87.2%), the combination of HE4 + CA125 the sensitivity and PPV reached 96.7% and 97% respectively.

**Conclusion:**

Measuring serum HE4 concentrations along with CA125 concentrations may provide higher accuracy for detecting epithelial ovarian cancer.

**Virtual Slides:**

The virtual slide(s) for this article can be found here: http://www.diagnosticpathology.diagnomx.eu/vs/1060413168685759

## Introduction

The annual incidence of ovarian cancer is 204,000, with 125,000 deaths. In developed countries, ovarian cancer remains the most lethal of all gynecologic malignancies. One of the reasons for the high fatality rate is that more than 70% of women with ovarian cancer are diagnosed with advanced disease. There is a close correlation between stage at presentation and survival; therefore, early detection of ovarian cancer represents the best hope for mortality reduction and long term disease control. There is preliminary evidence that screening can improve survival, but the impact of screening on mortality from ovarian cancer is still unclear [1].

Epithelial ovarian cancer set by the World Health Organization (WHO) recognizes eight histological tumor subtypes: serous, mucinous, endometrioid, clear cell, transitional cell, squamous cell, mixed epithelial and undifferentiated. Within each subtype, tumors are further described as benign, malignant, or borderline, and depending upon tumor subtype; classified as low or high-grade. Borderline tumors are considered to have low malignant potential and/or indolent behavior [2].

Serous tumors, which carry the poorest prognosis, are the most common form of ovarian carcinoma and make up 30-70% of all diagnoses. Serous tumors are histologically similar to cancers of the fallopian tube, and range from cystic papillary tumors to solid masses. Endometrioid tumors, accounting for 10-20% of ovarian carcinomas, are characterized by endometrial-like glandular structures. Mucinous tumors often contain cysts and glands lined by mucin-rich cells and constitute 5-20% of ovarian carcinomas. Clear cell tumors represent 3-10% of ovarian carcinomas and are comprised of clear and hobnailed cells with an immature glomerular pattern. Undifferentiated carcinomas constitute 1% [3].

Recent morphologic, immunohistochemical, and molecular genetic studies have led to the development of a new paradigm for the pathogenesis and origin of epithelial ovarian cancer based on a dualistic model of carcinogenesis that divides epithelial ovarian cancer into 2 broad categories designated types I and II. Type I tumors include all major histotypes (serous, endometrioid, mucinous, clear cell, and transitional) but exhibit low-grade nuclear and architectural features, slow growth, and can be linked to well-defined benign ovarian precursor lesions. They are generally indolent, present in stage I (tumor confined to the ovary), and are characterized by specific mutations, including KRAS, BRAF, ERBB2, CTNNB1, PTEN, PIK3CA, ARID1A, and PPP2R1A. Type II tumors comprise high-grade serous, high-grade endometrioid, malignant mixed mesodermal tumors (carcinosarcomas), and undifferentiated carcinomas. They are aggressive, present in advanced stage, and have a very high frequency of TP53 mutations and may also exhibit gene amplification and overexpression of HER2/neu and AKT2 oncogenes [4]. Several protooncogenes, tumor suppressor genes, and apoptosis related genes including bax, bcl-2, p53, p21, myc, c-kit, telomerase, and metallothionein have been investigated in ovarian tumors which can reliably predict the rate of progression and the response to chemotherapy and can facilitate ovarian cancer typing [5-8]. Recent Immunohistochemical staining of ovarian cancer for potassium channels (Kv1.3, K2p9.1, Eag and HERG) have been shown to be overexpressed in ovarian cancer where they appear to play a role in cell proliferation and progression [9].

Symptoms of epithelial ovarian cancer are often nonspecific, especially in early stage cancer. Ultrasound is used to assess patients for ovarian cancer; ultrasound has a low specificity for determining if a mass is benign or malignant. The specificity is improved by using Doppler ultrasound and a morphology index but performance varies amongst different operators [10].

The use of tumor markers to further characterize the mass has come into clinical use. Carbohydrate antigen 125 (CA125) is the most widely used tumor marker in ovarian cancer; however, its predictive power is far from ideal. It is elevated in about 80% of women with epithelial ovarian cancer (EOC) but only in 50% of women with early stage disease [11]. The specificity of CA125 is limited, since it can be elevated in a range of common benign gynecologic or non-gynecologic conditions [12]. Furthermore, the sensitivity and specificity of CA125 are not high enough for population screening for the detection of early stage ovarian cancer [13].

The identification of new cancer biomarkers to replace or complement CA125 is urgently needed and currently underway. Accordingly, there have been many efforts to improve the diagnostic performance of markers or marker combinations, and some markers, including mesothelin, CA72-4, inhibin, kallikreins, and osteopontin, have been investigated to complement CA125 and to improve its sensitivity for early detection [12,14]. Among these, human epididymis protein 4 (HE4), also known as WAP-type four disulphide core 2 (WFDC2), is one of the most promising markers for improving the sensitivity and specificity. HE4 is primarily expressed in the reproductive and respiratory tracts [15] and is overexpressed in ovarian cancer cells, especially in histologic subtypes of serous or endometriod carcinoma [16] and it has been suggested to be a serological marker of ovarian cancer [17]. In this study, we aimed to compare the characteristics of HE4 and CA125 in epithelial ovarian cancer and benign gynecological diseases, and to evaluate the diagnostic performance of both CA125 and HE4 in discriminating ovarian cancer from other benign gynecologic diseases.

## Methods

### Subjects and study design

In this study, 30 female patients were selected with recently diagnosed malignant epithelial ovarian cancers, who consecutively were admitted at Oncology department in Sohag University from March 2011 to July 2012. The inclusion criteria were: availability of complete clinical records, informed consent and agreement to have additional testing for new markers, clinical and histological diagnosis with staging and grading of ovarian cancer, according to the current classification and guidelines. The mean age was 50.7 ± 14.6 years (range 24-73), and 9 were in menopause (30%). The histological diagnosis was as follows: 21 serous (70%), 6 endometrioid (20%) and 3 mucinous tumors (10%). It was approved by the faculty committee for research ethics. The exclusion criteria: were pregnancy and significant concomitant diseases such as chronic heart failure, and severe chronic liver or renal disease. The second group consisted of 20 patients with benign gynecological diseases, with a mean age of 45.1 ± 14.7 years (range 40-60) and 5 in menopause (25%). The group with benign diseases included 8 patients with ovarian cyst (40%), 5 patients with endometriosis (25%), 4 patients with fibroid (20%) and 3 patients with pelvic inflammatory disease (15%). The third group included 20 apparently healthy females matched for the same age group as a control group.

### Methods

Venous blood samples were collected following an overnight fasting (serum and EDTA samples) before chemotherapy treatment and at 6 month intervals thereafter. Serum glucose, liver function tests and renal function testes were analyzed on autoanalyzer Cobas c 311 (Roche/Hitachi cobas C systems). Complete blood picture on cell dyne-2700 fully automated cell counter. CA125 analysis was done on Axsym system (Abbott Diagnostics Division, Chicago) based on Microparticle Enzyme Immunoassay (MEIA) technology. HE4 assay was performed on the fully automated ARCHITECT instrument (Abbott Diagnostics Division, Chicago) based on Chemiluminescentmicroparticle immunoassay (CMIA). The two monoclonal antibodies (2 H5 and 3D8) were used for capture and detection of HE4. In the first step, sample and 2H5 anti-HE4 coated paramagnetic microparticles are combined. HE4 antigen present in the sample binds to the anti-HE4 coated microparticles. After washing, 3D8 anti-HE4 acridinium labeled conjugate is added to create a reaction mixture in the second step. Following another wash cycle, pre-trigger and trigger solutions are added to the reaction mixture. The resulting chemiluminescent reaction is measured as relative light units. A direct relationship exists between the amount of HE4 antigen in the sample and the relative light units.

### Statistical analysis

The data are presented as mean ± standard deviation (SD) or median (range) and number (n). Linear relationships between variables were determined using Spearman’s rank correlation test. One way analysis of variance (ANOVA) test was used followed by post hoc test to determine the significance of variables when comparing more than 2 groups. Non-parametric receiver operating characteristic (ROC) analyses were performed to evaluate diagnostic values of individual parameters generated by graphically plotting sensitivity versus specificity with using 95% confidence intervals (CI). The diagnostic accuracy of the test is measured by the area under the curve (AUC). Statistical significance is considered a value of *P* <0.05. All statistical analyses were performed using SPSS software, version 10.0.

## Results

**Figure 1:**
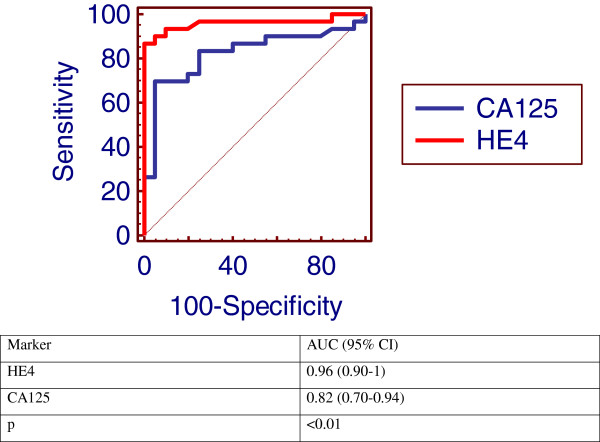
ROC plot and AUC for CA125 and HE4 for patients with epithelial ovarian cancer and benign diseases.

**Figure 2:**
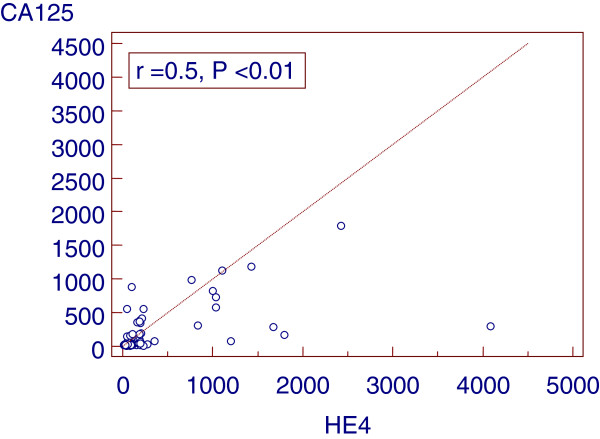
Correlation (Spearman) between HE4 and CA125 on all groups.

**Figure 3:**
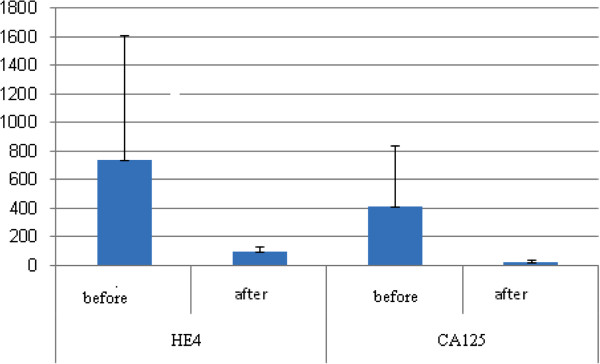
HE4 and CA125 in the ovarian cancer group before and after treatment.

Clinical characteristics and laboratory variables of the studied groups were demonstrated in Table 1. The median CA125 and HE4 levels in the healthy control were 12.5 U/ml and 56.9 pmol/l respectively. There were significant difference in HE4 & CA125 values between the ovarian cancer group (median 295.5 U/ml, for CA125, and median 237.2 pmol/l, for HE4) than the benign gynecological disease (median 26.9 U/ml, p = 0.001 for CA125, and median 66.1 pmol/l, p = 0.001 for HE4) and control group (p <0.001). CA125 was also higher in patients with benign gynecologic diseases (p = 0.04) than in the healthy control, but HE4 was not (p >0.05). Table 2 showed FIGO stage of the studied woman, 40% of the patients had stage 3 and the serum levels of HE4 and CA125 in relation to histological types with higher significant level in serous ovarian cancer (p <0.01 and p <0.05 respectively). In 30 women with EOC, HE4 were significantly higher than CA125 in sensitivity and specificity (90% vs 83.3% and 95% vs. 85%, respectively). Also, the, positive predictive values (PPV) and negative predictive values (NPV) for HE4 were significantly higher than CA125 (93.1% vs. 80.7% and 92.7% vs. 87.2%, respectively). Sensitivity and PPV were increased reached 96.7%, 97% respectively when the two markers combined with each other (Table 3). Figure 1 shows the receiver operating characteristic analysis (ROC) plot for all women with epithelial ovarian cancer and benign diseases, the area under the curve (AUC) for CA125 was 0.82 (95% CI 0.7-0.94) and for HE4 was 0.96 (95% CI 0.9-1.0) (p <0.01) for distinguishing between EOC and benign disease. A positive correlation between serum levels of HE4 and CA125 was observed in women with epithelial ovarian cancer, benign gynecological disease group and control group (r =0.5, p < 0.01) (Figure 2). Of the 30 patients in the study, three were resistant to treatment. The remaining 27 patients achieved remission determined by normalization of CA125 and HE4 levels, physical examination and imaging by computerized tomography (CT) magnetic resonance imaging (MRI) or ultrasound. Table 4 (Figure 3): show significant difference (P < 0.001) for CA125 and HE4 levels before and after chemotherapy. In the three patients with persistent disease following chemotherapy and the presence of tumor confirmed by imaging results (one patient with omental deposit and two patients with residual tissues), CA125 and HE4 dropped but remained above their respective thresholds thereafter.

**Table 1:** Clinical and laboratory variables of studied groups

	**Ovarian cancer (n = 30)**	**Benign gynecological (n = 20)**	**Controls (n = 20)**	**p (ANOVA)**
Age (range)	24-73	40-60	30-66	-
Menopause%	30	25	20	-
RBCs count x10^12^/l	4.5 ± 0.5	4.5 ±0.49	4.8 ± 0.5	NS
Hb g/dl	12.1 ± 1.5	12.5 ± 1.4	12 ± 1.3	NS
WBCs x10^9^/l	8.1 ± 2.9	6.9 ± 2.3	7.6 ± 2	NS
Platelets x10^9^/l	270 ± 82	294.7 ± 72.1	264.7 ± 59.4	NS
S.glucose (mg/dl)	95 ± 15.4	91 ± 7.4	85 ± 9.4	NS
S.urea (mg/dl)	31.4 ± 8.9	29.1 ± 6.7	27.9 ± 7.9	NS
S.creatinine (mg/dl)	0.7 ± 0.15	0.7 ± 0.13	0.73 ± 0.12	NS
ALT (U/l)	17.4 ± 12.6	15.4 ± 11.5	13.9 ± 7.2	NS
AST (U/l)	24.7 ± 14.5	21.1 ± 10.4	21.1 ± 6.1	NS
ALP (U/l)	76.4 ± 24.2	68.5 ± 10.9	70.5 ± 11.3	NS
Total protein g/dl	7.6 ± 0.6	7.4 ± 0.5	7.7 ± 0.53	NS
Albumin g/dl	3.8 ± 0.33	3.9 ± 0.36	3.9 ± 0.29	NS
Total bilirubin mg/dl	0.46 ± 0.3	0.6 ± 0.26	0.5 ± 0.24	NS
D. bilirubin mg/dl	0.12 ± 0.07	0.11 ± 0.06	0.1 ± 0.06	NS
CA125 U/ml	295.5 (4.2-1781)^b,c^	26.9 (9.4-553)^a^	12.5 (4.1-34.3)	<0.001
HE4 pmol/l	237.2 (34.3-4090)^b,c^	66.1 (24.8-179.2)	56.9 (20.8-111.6)	<0.001

Values are expressed as the mean ± standard deviation or median (range). ^a^P < 0.05, ^b^P < 0.001,with respect to the control group; ^c^P = 0.001, with respect to benign gynecological dis.

**Table 2:** HE4 & CA125 serum levels in patients with EOC according to tumor stage and histological type

	**n**	**HE4 median (range)**	**CA125 median (range)**
Stage I-II	8	210 (55-1060)	36 (4.2-410)
Stage III	12	315 (34.3-2817)	120 (26-1300)
Stage IV	10	491 (45-4090)	320(150-1781)
Serous	21	305 (57-4090)	285 (47-1781)
Endometrioid	6	170 (34.3-1650)	137 (35-1011)
Mucinous	3	159 (142-410)	66 (4.2-405)

**Table 3:** Sensitivity, specificity, positive and negative predictive values (PPV and NPV) for CA125, HE4, and in combination with each other

	**Sensitivity**	**Specificity**	**PPV**	**NPV**
HE4	90%	95%	93.1%	92.7%
CA125	83.3%	85%	80.7%	87.2%
HE4 + CA125	96.7%	80%	97%	80%

**Table 4:** Levels of HE4 and CA125 in the ovarian cancer group before and after treatment

	**Before treatment**	**After treatment**	**P value**
**HE4**	**237.2 (34.3-4090)**	**91.9 (45.4-180)**	**<0.001**
**CA125**	**295.5 (4.2-1781)**	**11.6 (4-71.5)**	**<0.001**

## Discussion

Despite the development of new treatments and therapies designed to improve the five year survival rate, ovarian cancer still remains the deadliest cancer of the female reproductive tract. Five-year survival rate is 90% when disease is confined to the ovaries but overall survival is poor because only 25% of cases are found in this early stage. Unfortunately, most cases are diagnosed in the late stages of the disease, when the five-year survival rates fall below 20%, with most patients having metastatic disease at presentation. This further contributes to worsening the prognosis. The lack of precise early warning signs is one of the factors that further contribute to the fact that only 25% of ovarian tumors are identified at stage I [18].

CA125 is still the only tumor marker recommended as a diagnostic or prognostic indicator and for the monitoring of disease recurrence after surgery and adjuvant chemotherapy [19-21]. The major drawback of CA125 is the documented lack of specificity, as this marker may show levels exceeding the 95th percentile of normal values in a significant proportion of women with benign or malignant diseases [22].

Accordingly, there have been many efforts to improve the diagnostic performance of CA125. Among those, a prominent relevance has been recently attributed to the HE4 which is one of the most promising marker for improving the sensitivity and specificity [23].

In this study, we investigated the role of HE4 alone and in combination with CA125 in assessing patients with epithelial ovarian cancer, regardless of the menopausal status. Initial results on HE4 testing of this study confirm the high sensitivity and specificity of this molecule over CA125 for EOC (90% vs. 83.3% and 95% vs. 85%, respectively). In our experience, no false positive results for HE4 or CA125 were recorded on healthy women, the specificity was more evident among patients with benign gynecological lesions, since only 2 were positive for HE4, while 6 were positive for CA125 (10% vs 30%). we found a significant difference in CA125 values between the benign gynecological disease group and control group (p < 0.05). Unlike CA125, HE4 was shown not to be elevated in endometriosis [24], [25] and this may contribute to the improved performance with HE4.

The diagnostic performance of CA125 and HE4 in discriminating ovarian cancer from healthy and benign gynecologic conditions was verified using ROC analysis. The resultant AUC values were 0.96 for HE4 (95% CI 0.90-1.0) and 0.82 for CA125 (95% CI 0.70-0.94) (p < 0.01), which would make them feasible for use as tumor markers, have also investigated the usefulness of HE4 in to differentiate ovarian cancers from healthy and benign gynecologic conditions. This finding is in agreement with several researchers who reported that HE4 has a clear specificity edge over CA125 and also a better sensitivity for EOC, in general and in the early stages patients [26-30].

HE4 as well as CA125 are not only found in ovarian carcinoma; abnormal levels may be also found in some benign conditions or other gynecologic and non-gynecologic malignancies; for example, breast, pancreatic, and endometrial cancers. These results imply that HE4 and CA125 are not especially specific to ovarian cancer [31].

Another study demonstrated that HE4 might be associated with the innate immune defenses of the lung, nasal and oral cavities [32]. Several researchers have also investigated the usefulness of HE4 in other malignancies, including transitional cell carcinoma of the urinary tract and endometrial cancer [33].

The limits of any single tumor marker for EOC have been addressed in several recent experiences, in which a multimarker approach has been pursued in order to achieve a better diagnostic accuracy [34-38]. Combined the two markers showed improved sensitivity to 96.7%, and increased PPV. There was a 12.4% increase in PPV for CA125 compared to HE4 (16.3% vs. 3.9%). Improving the PPV not only means less inappropriate referrals and its associated costs, but also a reduction in the number of midline laparotomies, which is still the standard for cares for women with suspected EOC.

The correlation between HE4 and CA125 levels was estimated using Spearman’s rank correlation test, most of the measured values tended to be increased for both markers. However, the degree of correlation was not so strong (r = 0.5), and there were some discordant results. These mean that each marker was elevated concurrently or under some different conditions, and these also support the necessity of combining the two markers.

The increases in both markers were more evident in certain histologies of ovarian cancer than in others. Galgano et al. reported that HE4 proteins or genes were expressed strongly in serous papillary, clear cell, and endometrioid carcinoma of the ovaries. However, other histologies of ovary cancer or non-gynecologic malignancies including invasive ductal carcinoma of breast, endometrial, pancreaticobiliary, and renal cell carcinoma also exhibited strong or weak expressions of HE4 proteins [15]. In our result, the predominant histological type observed is serous followed by endometriod and mucinous, we found both tumor markers HE4 and CA125 were related to tumor stage and histological types with the lowest concentration in mucinous subtype (median 66 U/ml, for CA125, and median 159 pmol/l, for HE4) and elevation of HE4 or CA125 was obvious in serous subtypes (median 285 U/ml, for CA125, and median 305 pmol/l, for HE4) but was not evident in other histological type. Kobel et al. investigated the variation in expression of 21 different markers in accordance with ovarian cancer subtypes, and concluded that most markers differ significantly between histological types [39].

In assessment of treatment response both CA125 and HE4 levels show significant difference before and after chemotherapy (P < 0.001) for both, in which normalization of CA125 and HE4 levels occurred. Relevant thresholds for using these markers for remission monitoring have not been established. There is an established threshold range for various commercial CA125 assays [40]. Thresholds for HE4 have been reported only recently but remain uncertain [41-43]. In three patients CA125 and HE4 dropped but remained above their respective thresholds thereafter (one patient had high CA125 and others had high HE4). HE4 serum levels are related to progression of disease stage [44] and hence to tumor burden. A failure of HE4 levels to normalize at the completion of primary therapy could be related to persistent disease not detected by CA125 nor by physical exam or CT imaging. These patients may represent a high risk group who could potentially benefit from additional treatment or more intensive monitoring. Confirmation of this HE4 behavior in a larger number of patients is therefore required.

In addition to the search of specific markers for the determination of the early stages of any cancer form, it is just as important to find markers capable of following the remission from disease as response to therapy. The suggestion that HE4 is a good indicator for the remission from the disease was reported by a follow up study by Allard et al. in which it was shown that the values of HE4 correlated with the clinical response to treatment or remission from the disease, as documented by CT imaging [45].

## Conclusion

HE4 demonstrated comparable diagnostic performances to CA125 as a tumor marker for detecting ovarian cancer. HE4 was more sensitive in detecting early stages of ovarian cancer and more specific. HE4 improves the utility of CA125 as a tumor marker in ovarian cancer, and using both markers simultaneously increases the tumor marker sensitivity. The use of this combination might enable to improve detection of ovarian cancer as compared with use of either marker alone for the discrimination of benign from malignant ovarian lesions.

## Abbreviations

HE4: Human epididymis protein 4; CA125: Carbohydrate antigen125; EOC: Epithelial ovarian cancer; PPV: Positive predictive value; NPV: Negative predictive value; ROC: Receiver operating characteristic analysis; AUC: Area under the curve; RBCs: Red blood cells; WBCs: White blood cells; ALT: Alanine transaminase; AST: Aspartate transaminase; ALP: Alkaline phosphatase

## Competing interest

The authors declare that they have no conflict of interest and did not receive any financial support from any source outside the university.

## Authors’ contributions

I and my Clinical Pathology colleagues have tested the serum markers either CA125 or HE4 besides routine investigation and prepared the introduction, results and discussion of this article. My colleague from the Obstetrics & Gynecology department has provided us with the specimens from patients and shared in the discussion of this article. My colleague from the clinical oncology department has made and commented on the clinical, histological diagnosis with staging and grading of ovarian cancer, CT examination of the patient and chemotherapy treatment. All authors read and approved the final manuscript.
